# *S. aureus* Evades Macrophage Killing through NLRP3-Dependent Effects on Mitochondrial Trafficking

**DOI:** 10.1016/j.celrep.2018.02.027

**Published:** 2018-02-27

**Authors:** Taylor S. Cohen, Michelle L. Boland, Brandon B. Boland, Virginia Takahashi, Andrey Tovchigrechko, Young Lee, Aimee D. Wilde, Mark J. Mazaitis, Omari Jones-Nelson, Christine Tkaczyk, Rajiv Raja, C. Kendall Stover, Bret R. Sellman

**Affiliations:** 1 Department of Infectious Disease, MedImmune LLC, Gaithersburg, MD, USA; 2 Department of Cardiovascular and Metabolic Disease, MedImmune LLC, Gaithersburg, MD, USA; 3 Department of Translational Medicine and Pharmacogenomics, MedImmune LLC, Gaithersburg, MD, USA; 4 Department of Pathology, Microbiology, and Immunology, Vanderbilt University Medical Center, Nashville, TN, USA; 5 Nascent Studio, Ann Harbor, MI, USA; 6 Lead Contact

## Abstract

Clinical severity of *Staphylococcus aureus* respiratory infection correlates with alpha toxin (AT) expression. AT activates the NLRP3 inflammasome; deletion of *Nlrp3*, or AT neutralization, protects mice from lethal *S. aureus* pneumonia. We tested the hypothesis that this protection is not due to a reduction in inflammasome-dependent cytokines (IL-1β/IL-18) but increased bactericidal function of macrophages. *In vivo*, neutralization of AT or NLRP3 improved bacterial clearance and survival, while blocking IL-1β/IL-18 did not. Primary human monocytes were used *in vitro* to determine the mechanism through which NLRP3 alters bacterial killing. In cells treated with small interfering RNA (siRNA) targeting NLRP3 or infected with AT-null *S. aureus*, mitochondria co-localize with bacterial-containing phagosomes. Mitochondrial engagement activates caspase-1, a process dependent on complex II of the electron transport chain, near the phagosome, promoting its acidification. These data demonstrate a mechanism utilized by *S. aureus* to sequester itself from antimicrobial processes within the cell.

## Graphical Abstract

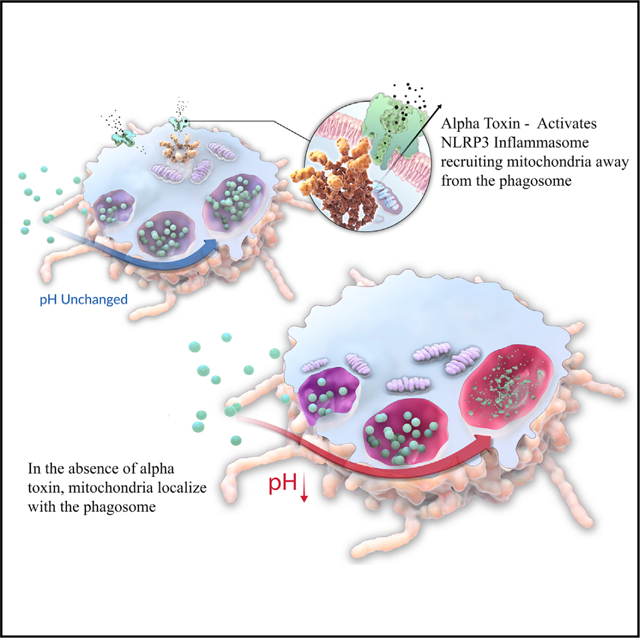

## In Brief

In the lung, alpha toxin (AT) is a primary virulence factor used by *S. aureus* to evade innate immune responses. Cohen et al. demonstrate that AT activation of the NLRP3 inflammasome uncouples key components of the phagocytic killing machinery, namely, mitochondria dissociate from internalized bacteria. Without close association of mitochondria with internalized bacteria, macrophages are unable to effectively kill *S. aureus*.

## INTRODUCTION

*Staphylococcus aureus* is a major human pathogen commonly found colonizing the nasopharynx but capable of infecting the lower airways. A member of the ESKAPE pathogen family (*Enterococcus faecium*, *S. aureus*, *Klebsiella pneumoniae*, *Acinetobacter baumannii*, *Pseudomonas aeruginosa*, and *Enterobacter* species), *S. aureus* has developed resistance to many commonly used antibiotics, and even serious antibiotic-susceptible *S. aureus* infections can cause significant morbidity and mortality (Parker et al., 2016). To circumvent antimicrobial resistance and minimize broad-spectrum antibiotic impact on the beneficial microbiota, alternate strategies for combating these pathogens, such as enhancement of beneficial host defense components, are required. Development of new therapeutic strategies necessitates a deeper understanding of the mechanisms used by *S. aureus* to evade innate immune clearance.

*S. aureus* utilizes a vast array of virulence factors to mask itself from immune detection, modulate host immune signaling, or directly lyse host cells. In respiratory infections, the expression of alpha toxin (AT) has been correlated with worse clinical outcome, and observations supported by murine and rabbit models of infection demonstrate that AT does, in fact, significantly contribute to the mortality and morbidity associated with respiratory infections (Arroliga et al., 2014; Bonesso et al., 2016; Diep et al., 2016; Inoshima et al., 2011; Stulik et al., 2014). Because of its contribution to respiratory disease and that approximately 99% of *S. aureus* isolates contain the AT gene, as compared with other relevant toxins such as Panton Valentine Leucocidin (PVL, ~20% of isolates), AT targeting is being actively pursued for the treatment or prevention of *S. aureus* pneumonia (Hua et al., 2014; Rouha et al., 2015; Sause et al., 2016; Tabor et al., 2016).

At the cellular level, AT binds to the receptor, a disintegrin and metalloproteinase domain-containing protein 10 (ADAM10), where it oligomerizes and forms a pore in the cell membrane, enabling free ion flow into and out of the cell (Inoshima et al., 2011). At high concentrations, AT lyses the targeted cell; but, at low concentrations, pore formation can activate cellular pathways, including calpain signaling and the inflammasome, both of which are thought to influence innate immune defenses (Cohen et al., 2016; Kebaier et al., 2012). Potassium flux through the AT pore activates the NLRP3 inflammasome (Craven et al., 2009). In response to altered potassium levels, NLRP3 complexes with ASC (*Pycard*), in turn cleaving pro-caspase-1 into caspase-1, which then cleaves pro-IL-1β and pro-IL-18 prior to release from the cell. Expression of mature IL-1β and IL-18 has been correlated with impairment in host defense, and NLRP3-null mice are reportedly protected from *S. aureus* pneumonia (Kebaier et al., 2012).

Somewhat paradoxically, recent work suggested that activation of caspase-1 is required for the processing and killing of *S. aureus* (Sokolovska et al., 2013). We hypothesized that AT was activating caspase-1 via a different mechanism than that required for bacterial killing. Our data show that, in response to *S. aureus* AT, mitochondria preferentially associate with the inflammasome, forming a complex that leads to robust caspase-1 activation and induction of downstream signaling. However, in cells infected with bacteria lacking AT, mitochondria co-localize with phagocytosed *S. aureus*. These mitochondria alter their electron transport chain, resulting in increased reactive oxygen species (ROS) generation, activation of caspase-1 locally, and initiation of endosomal acidification. We conclude that AT protects *S. aureus* from macrophage killing by spatially disengaging mitochondria from internalized bacteria, and neutralization of this toxin protects against respiratory infection not by limiting cytokine production but rather by enabling mitochondrial and bacterial co-localization within the cell.

## RESULTS

### Neutralization of AT or NLRP3 Inhibition Provides Greater Protection than IL-1β/IL-18 Neutralization

**Figure 1.**
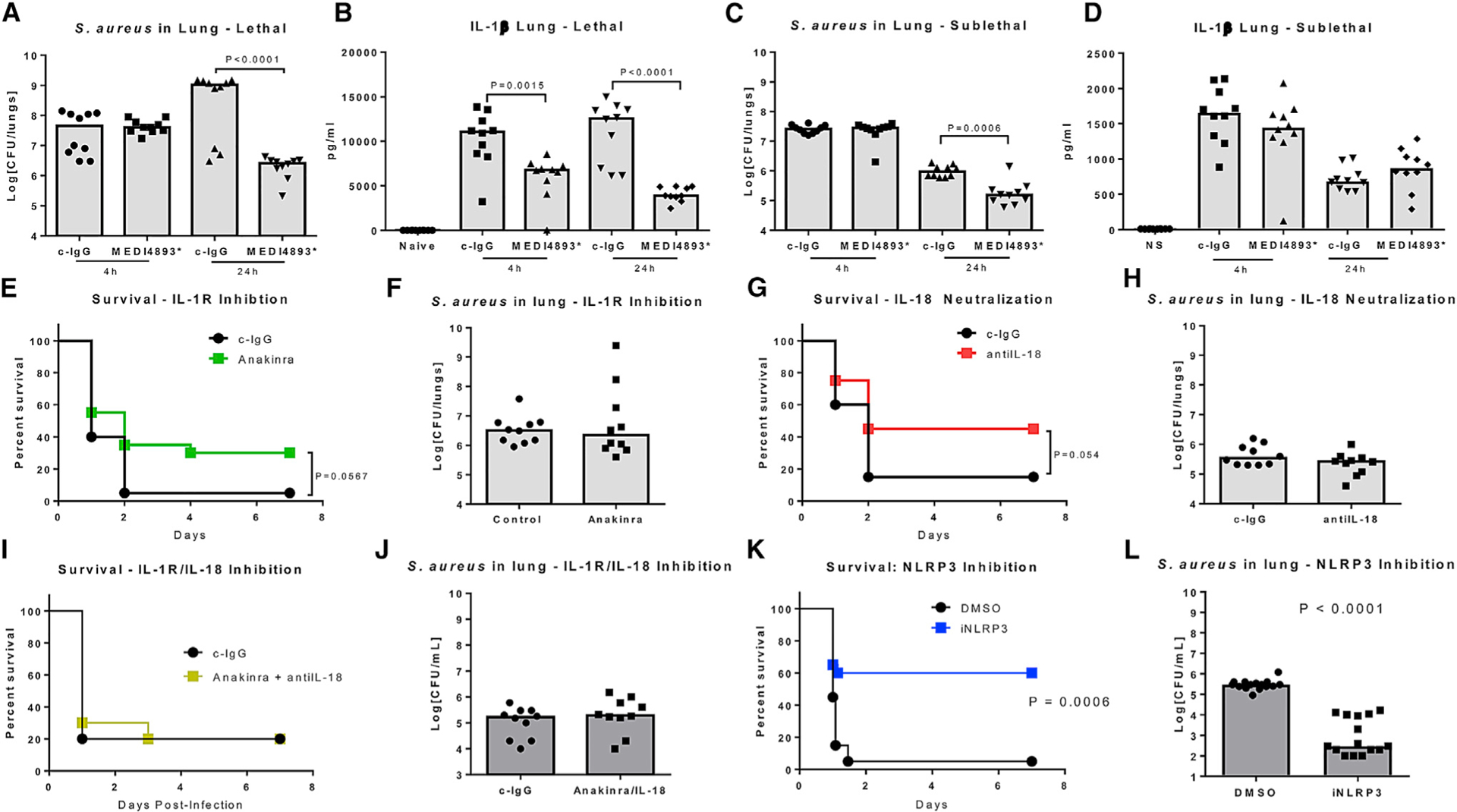
AT and NLRP3 Inhibition Promotes Bacterial Clearance (A) *S. aureus* CFUs recovered from the lungs of infected (1e8 CFUs) mice treated with MEDI4893* (15 mg/kg) or c-IgG 24 hr prior to infection. (B) IL-1β levels in BALF 4 and 24 hr following infection (1e8 CFUs) in mice treated with MEDI4893* (15 mg/kg) or c-IgG 24 hr prior to infection. (C) *S. aureus* CFUs recovered from the lungs of infected (5e7 CFUs) mice treated with MEDI4893* (15 mg/kg) or c-IgG 24 hr prior to infection. (D) IL-1β levels in BALF 4 and 24 hr following infection (5e7 CFUs) in mice treated with MEDI4893* (15 mg/kg) or c-IgG 24 hr prior to infection. (E) Survival of mice treated with Anakinra or c-IgG and infected with 1e8 CFUs *S. aureus*. (F) *S. aureus* CFUs recovered from the lungs of infected (5e7 CFUs) mice treated with Anakinra or c-IgG 24 hr prior to infection. (G) Survival of mice treated with anti-IL-18 mAb or c-IgG and infected with 1e8 CFUs *S. aureus*. (H) *S. aureus* CFUs recovered from the lungs of infected (5e7 CFUs) mice treated with anti-IL-18 mAb or c-IgG 24 hr prior to infection. (I) Survival of mice treated with both Anakinra and anti-IL-18 mAb, or c-IgG and infected with 1e8 CFUs *S. aureus*. (J) *S. aureus* CFUs recovered from the lungs of infected (5e7 CFUs) mice treated with both Anakinra and anti-IL-18 mAb, or c-IgG 24 hr prior to infection. (K) Survival of mice treated (1 hr prior to infection) with MCC950 or vehicle control (DMSO) and infected with 1e8 CFUs *S. aureus*. (L) *S. aureus* CFUs recovered from the lungs of infected (5e7 CFUs) mice treated (1 hr prior to infection) with MCC950 or vehicle control (DMSO). Statistical significance was determined by (A–D, F, H, J, and L) Mann-Whitney test or (E, G, I, and K) log-rank test. All data are representative of greater than 2 independent experiments and n ≥ 10 mice per group per experiment.

The role of caspase-1 in the killing of *S. aureus*, if *Nlrp3*^−/−^ mice are protected from infection with this pathogen, is unclear. We hypothesized that if inflammasome-driven cytokines were contributing to *S. aureus* infection, cytokine levels would correlate with bacterial clearance. We began by infecting mice with either lethal (1e8 colony-forming unit [CFU]) or sublethal (5e7 CFU) numbers of *S. aureus* in the presence of AT-neutralizing monoclonal antibody (mAb) MEDI4893* and monitoring bacterial clearance and IL-1β levels in the airway 4 and 24 hr post-infection. Neutralization of AT resulted in a significant reduction in bacterial numbers recovered from the lungs of mice receiving either dose of bacteria (Figures 1A and 1C), but it only led to a reduction of IL-1β in mice given a lethal dose of *S. aureus* (Figures 1B and 1D). These data suggest that inflammasome-dependent cytokine production, a direct result of AT activity, might only correlate with survival and does not impair bacterial clearance. Unlike MEDI4893*, which protected 100% of the mice from lethal pneumonia, neutralization of the individual cytokines, IL-1 or IL-18, alone or in combination did not significantly affect survival during a lethal infection or bacterial clearance in a sublethal infection (Figures 1E–1J; Figure S1). Neutralization of the inflammasome with the small molecule MCC950 resulted in significant increases in survival (p = 0.0006) and bacterial clearance (p < 0.0001) (Figures 1K and 1L) (Coll et al., 2015). We concluded that AT neutralization, or direct targeting of NLRP3, provides protection independently from the activities of downstream cytokines.

### Bactericidal Capacity of Macrophages Is Dependent on Caspase-1

Protein levels in bronchoalveolar lavage (BAL) fluid of mice sublethally infected with *S. aureus* was not influenced by the administration of MEDI4893* (Figure S2A), and at these challenge innocula levels bacteria are not observed in the spleen (Figure S2B), indicating that AT was damaging the epithelial barrier and bacteria were not disseminating systemically at this innocula. We have previously reported that AT preferentially targets airway macrophages due to high expression levels of ADAM10; we therefore chose to focus on the effect of AT on macrophage function (Cohen et al., 2016). Alveolar macrophages were purified from BAL of naive mice or mice pretreated with MEDI4893* or c-IgG and infected with 5e7 CFU *S. aureus* for 24 hr, and gene expression was analyzed by microarray. Significant alterations in gene expression were observed in cells isolated from infected mice as compared with naive (Figures S2C–S2E). Neutralization of AT did not influence the transcriptional response of alveolar macrophages at this time point, indicating that AT does not influence macrophage function at the level of gene expression.

**Figure 2.**
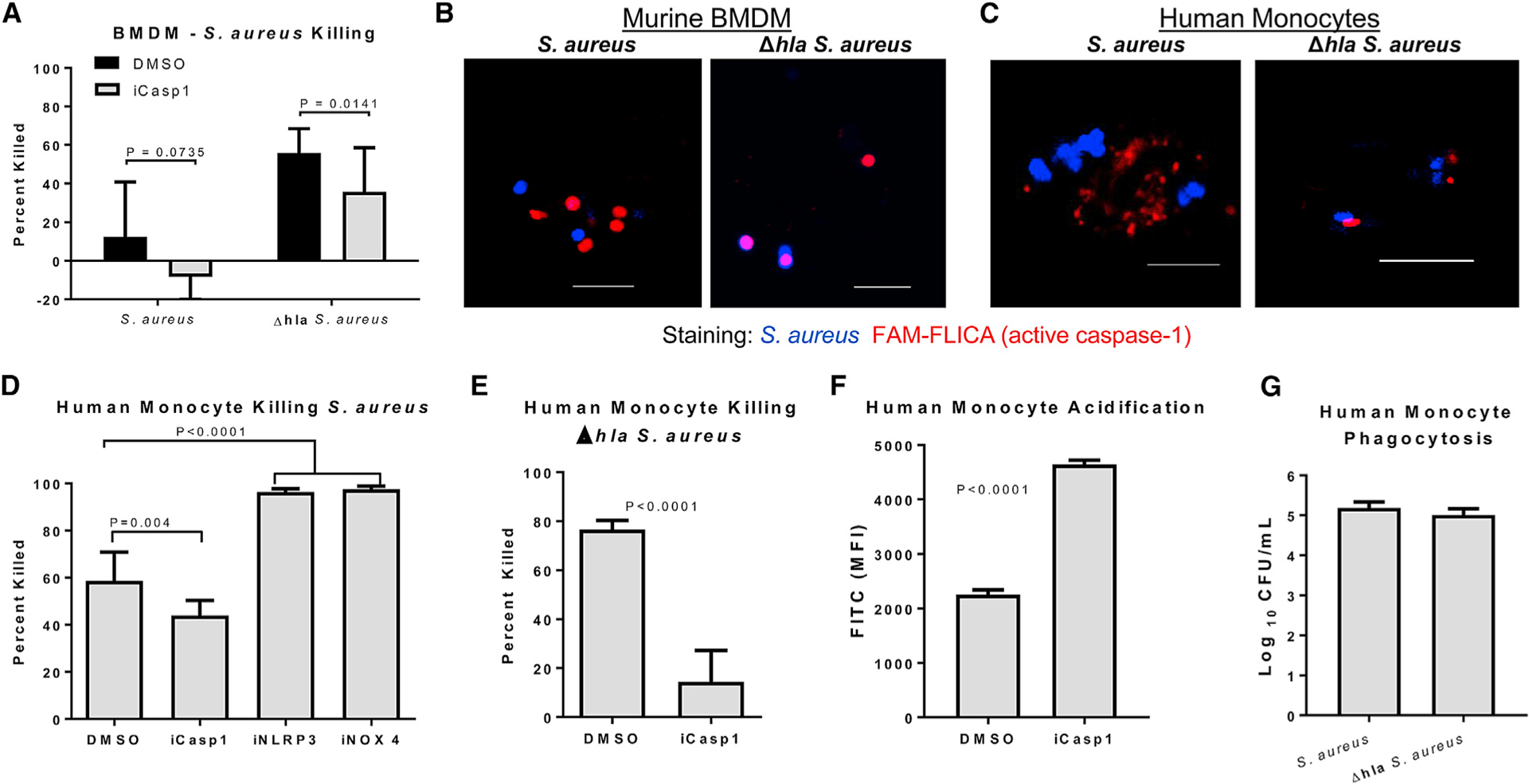
Caspase-1 Is Required for the Killing of *S. aureus* (A) Percentage of WT or Δ*hla S. aureus* killed following 1-hr incubation with BMDMs (MOI 1) following treatment with caspase-1 inhibitor or DMSO. (B) Confocal images of active caspase-1 (FAM-FLICA, red) and WT or Δ*hla S. aureus*(blue) withinlive BMDMs following 1-hr incubation with the bacteria. Scale bar, 5 mm. (C) Confocal images of active caspase-1 (FAM-FLICA, red) and WT or Δ*hla S. aureus* (blue) within live primary human monocytes following 1-hr incubation with the bacteria. Scale bar, 5 mm. (D) Percentage of *S. aureus* killed following 1-hr incubation (MOI 1) with primary human monocytes treated with caspase-1 inhibitor, MCC950 (iNLRP3), GKT127831 (iNOX4), or DMSO. (E) Percentage of Δ*hla S. aureus* killed following 1-hr incubation (MOI 1) with primary human monocytes treated with caspase-1 inhibitor or DMSO. (F) Fluorescence-activated cell sorting (FACS) analysis of acidification of the bacterial microenvironment in primary human monocytes treated with caspase-1 inhibitor or DMSO and infected with Δ*hla S. aureus*. (G) Numbers of *S. aureus* phagocytosed by human monocytes over a 1-hr incubation period. Statistical significance was determined by Mann-Whitney test or (D) ANOVA followed by Dunn’s test. All data are representative of 3 independent experiments. (A and D–G) n ≥ 4 replicates per group. Data presented as mean ± SD.

To better understand the influence of AT on macrophage killing, we tested the ability of murine bone marrow-derived macrophages (BMDMs) to kill wild-type (WT) or Δ*hla S. aureus*. As previously reported, killing of WT bacteria was significantly reduced (p = 0.0024) as compared with killing of Δ*hla S. aureus* (Figure 2A) (Cohen et al., 2016). Increased killing was not due to a difference in phagocytosis, as similar numbers of WT and Δ*hla S. aureus* were recovered from within BMDMs following a 30-min incubation followed by gentamicin killing of extracellular bacteria (Figure S3A). Sokolovska et al. (2013) demonstrated that the killing of *S. aureus* correlated with caspase-1 co-localization with internalized bacteria. Live-cell imaging confirmed that AT expression and bacterial killing correlated with *S. aureus* co-localization with activated caspase-1 (FAM-FLICA) (Figure 2B). As reported previously, caspase-1 inhibition impaired killing of Δ*hla S. aureus* (p = 0.0141), not the WT strain (Figure 2A), and prevented acidification of the Δ*hla S. aureus* microenvironment (Figure S3B), confirming its role in endosomal acidification and killing of *S. aureus*.

AT activates caspase-1 via the NLRP3 inflammasome, which forms a protein complex in the cytoplasm along with the adaptor protein apoptosis-associated speck-like protein containing a CARD (ASC or PYCARD) (Schroder and Tschopp, 2010). Due to enhanced caspase-1 co-localization with Δ*hla S. aureus*, we hypothesized that AT-driven inflammasome activation was sequestering caspase-1 away from the bacteria-containing phagosome. To test this, we treated BMDMs with small interfering RNA (siRNA) against NLRP3 or ASC, then measured the ability of these cells to kill *S. aureus*, and monitored the localization of cleaved caspase-1 and internalized bacteria. Significant increases in bacterial killing were observed in BMDMs treated with either NLRP3 or ASC siRNA as compared with scrambled siRNA (Figure S3C). Cells treated with scrambled siRNA control had large amounts of activated caspase-1 throughout their cytoplasm, while NLRP3 and ASC siRNA-treated cells had very localized caspase-1 staining that was in close proximity to the internalized bacteria (Figure S3D).

### *S. aureus* Killing by Human Monocytes Is Also Dependent on Caspase-1

Human monocytes, as opposed to murine, are sensitive to additional *S. aureus* toxins, such as Panton-Valentine leucocidin (PVL), and they can utilize an alternate pathway for inflammasome activation, in addition to the classical inflammasome activation pathway involving receptor-interacting serine/threonine kinase 3 (RIPK3) (DuMont and Torres, 2014; Gaidt et al., 2016). Therefore, we tested whether there was a similar AT dependency on caspase-1 activation in human cells. Human monocytes were purified from the blood of healthy donors and stimulated with WT or Δ*hla S. aureus*. Caspase-1 activation was measured by FAM-FLICA staining. Activation of caspase-1 was observed following a 1-hr incubation with either bacteria, however, induction was reduced in Δ*hla S. aureus-*treated cells (Figure S3E). ASC specks were also absent in Δ*hla S. aureus-*treated macrophages, indicating a lack of inflammasome complex formation (Figure S3F). Visualization of active caspase-1 within human monocytes demonstrated a close association with internalized Δ*hla S. aureus*, while staining was dispersed throughout cells infected with WT *S. aureus* (Figure 2C).

As similar AT-dependent caspase-1 activation and localization was observed in human cells as compared with murine, we hypothesized that caspase-1 inhibition would also influence bacterial killing by primary human monocytes. Neutralization of caspase-1 significantly reduced the killing of both WT (p = 0.004) and Δ*hla* (p < 0.0001) *S. aureus*, and it prevented acidification of the bacterial microenvironment; however, the effect of caspase-1 neutralization was much more pronounced in Δ*hla S. aureus* as observed in murine BMDMs (Figures 2D–2F). Phagocytosis was not influenced by the presence of AT (Figure 2G). We conclude that the increased effect of caspase-1 inhibition in Δ*hla S. aureus*-infected cells is a reflection of the increased association of caspase-1 with bacteria in these cells as compared with WT *S. aureus*. AT influences caspase-1 activation through the NLRP3 inflammasome; therefore, we took advantage of the potent, small molecule inhibitor of the NLRP3 inflammasome (MCC950) and demonstrated that NLRP3 inhibition significantly improved the killing (p < 0.0001) of WT *S. aureus*, confirming the results gathered from murine BMDM studies (Figure 2D). These data suggest that NLRP3 and caspase-1 in murine and human cells play similar roles in the killing of *S. aureus*.

Activation of the NLRP3 inflammasome has been shown to depend on cellular metabolism, specifically through the activity of NADPH oxidase 4 (NOX4), a major source of mitochondrial ROS (Kuroda et al., 2010; Moon et al., 2016). Originally identified in response to LPS, NOX4 has now been shown to mediate NLRP3 activation in response to Gram-positive *Streptococcus pneumoniae*; therefore, we tested the effect of NOX4 inhibition on the killing of *S. aureus* by human monocytes (Moon et al., 2016; Park et al., 2004). NOX4 inhibition significantly increased killing (p < 0.0001) of *S. aureus* by human monocytes, further correlating NLRP3 activation with the inhibition of *S. aureus* killing (Figure 2D).

### AT Disrupts Mitochondrial ROS-Driven Killing of *S. aureus*

**Figure 3.**
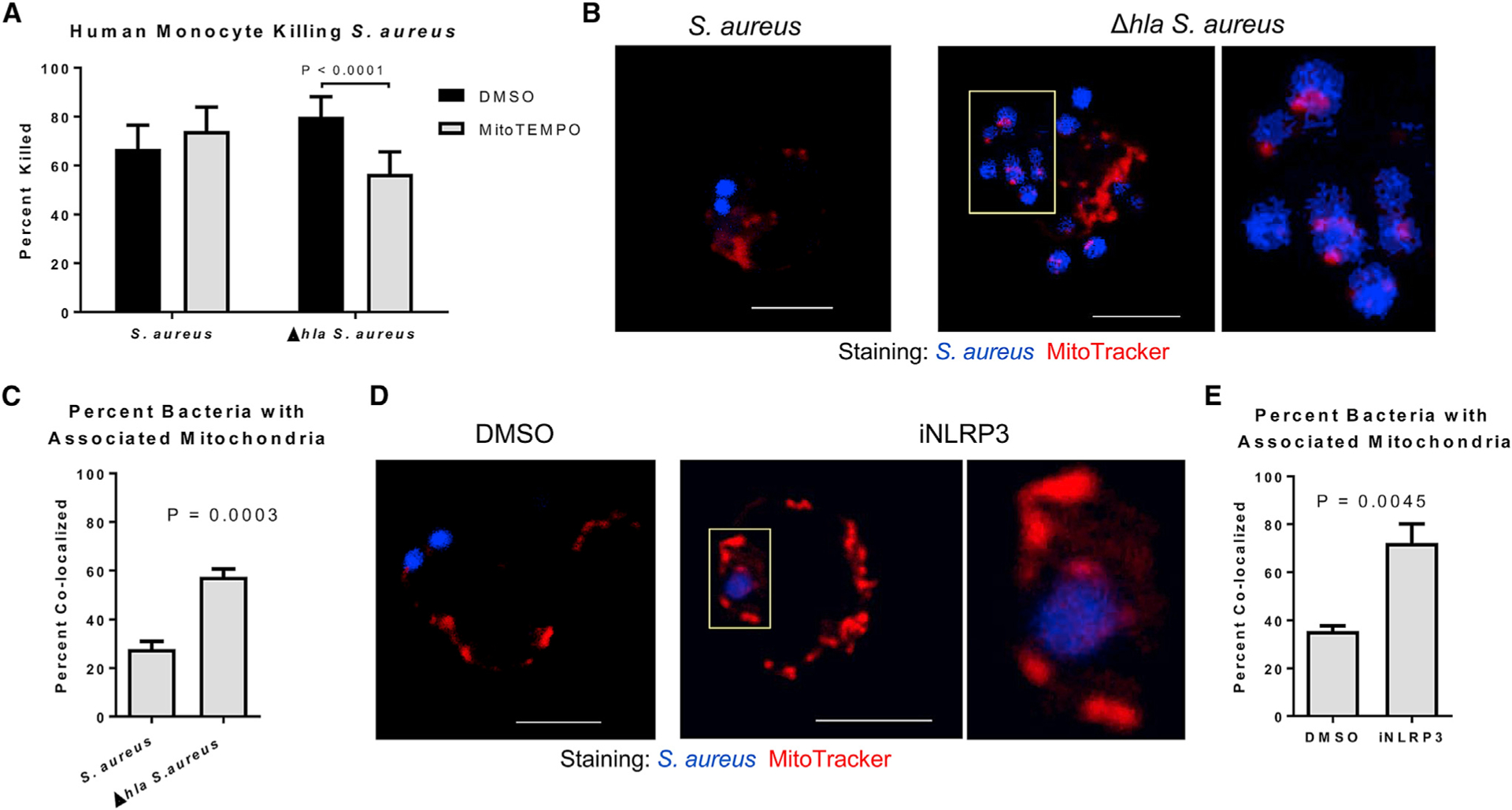
Mitochondria Significantly Contribute to the Killing of Δhla *S. aureus* (A) Percentage of WT or Δ*hla S. aureus* killed following 1-hr incubation with mitoTEMPO- or DMSO-treated (30 min) primary human monocytes. (B) Confocal images of mitochondria (MitoTracker) and WT or Δ*hla S. aureus* within live primary human monocytes. Scale bar, 5 μm; yellow box indicates the magnified area. (C) Quantification of the percentage of internalized WT or Δ*hla S. aureus* associated with a mitochondria. n > 100 combined from 3 individual experiments. (D) Confocal images of mitochondria (MitoTracker) and WT *S. aureus* within live primary human monocytes treated with MCC950 or DMSO. Scale bar, 5 μm; yellow box indicates the magnified area. (E) Quantification of the percentage of internalized *S. aureus* associated with a mitochondria in cells treated with MCC950 or DMSO. n > 50 combined from 3 individual experiments. Statistical significance (A, C, and E) was determined by Mann-Whitney test. All data are representative of 3 independent experiments. (A) n ≥ 4 replicates per group. Data presented as mean ± SD.

Shifts in the electron transport chain can lead to increased mitochondrial ROS, caspase-1 activation, and IL-1β cleavage and release (Garaude et al., 2016; Mills et al., 2016; Zhou et al., 2011). Both WT and Δ*hla S. aureus* induced mitochondrial ROS production and caspase-1 activation, which was reduced in both groups by treatment with the mitochondrial-specific ROS scavenger, mitoTEMPO (Figures S4A and S4B). Utilizing mitoTEMPO, we demonstrated that the killing of Δ*hla S. aureus*, but not WT *S. aureus*, by human monocytes was dependent on mitochondrial ROS (Figure 3A). Visualization of mitochondria within live monocytes infected with either *S. aureus* or Δ*hla S. aureus* revealed that significantly (p = 0.0003) more internalized Δ*hla S. aureus* was associated with mitochondria than internalized WT *S. aureus* (Figures 3B and 3C). Live-cell imaging was confirmed by electron microscopy (Figure S4C). Treatment of monocytes with MCC950 prior to infection with WT *S. aureus* resulted in significantly increased (p = 0.0045) co-localization of mitochondria and internalized bacteria (Figures 3D and 3E). Mitochondrial tracking to the phagosome is mediated by the activation of the kinases Mst1 and Mst2 (Geng et al., 2015). siRNA targeting of these kinases significantly impaired the ability of monocytes to kill both WT (p = 0.0233) and Δ*hla* (p = 0.0403) *S. aureus*, suggesting these kinases contribute to *S. aureus* killing (Figure S4D). We conclude that inflammasome activation in response to AT limits mitochondrial localization with phagocytosed bacteria, thereby reducing the bacterial killing capacity of the cell.

### Bactericidal Activity Is Dependent on ETC Complex II

**Figure 4.**
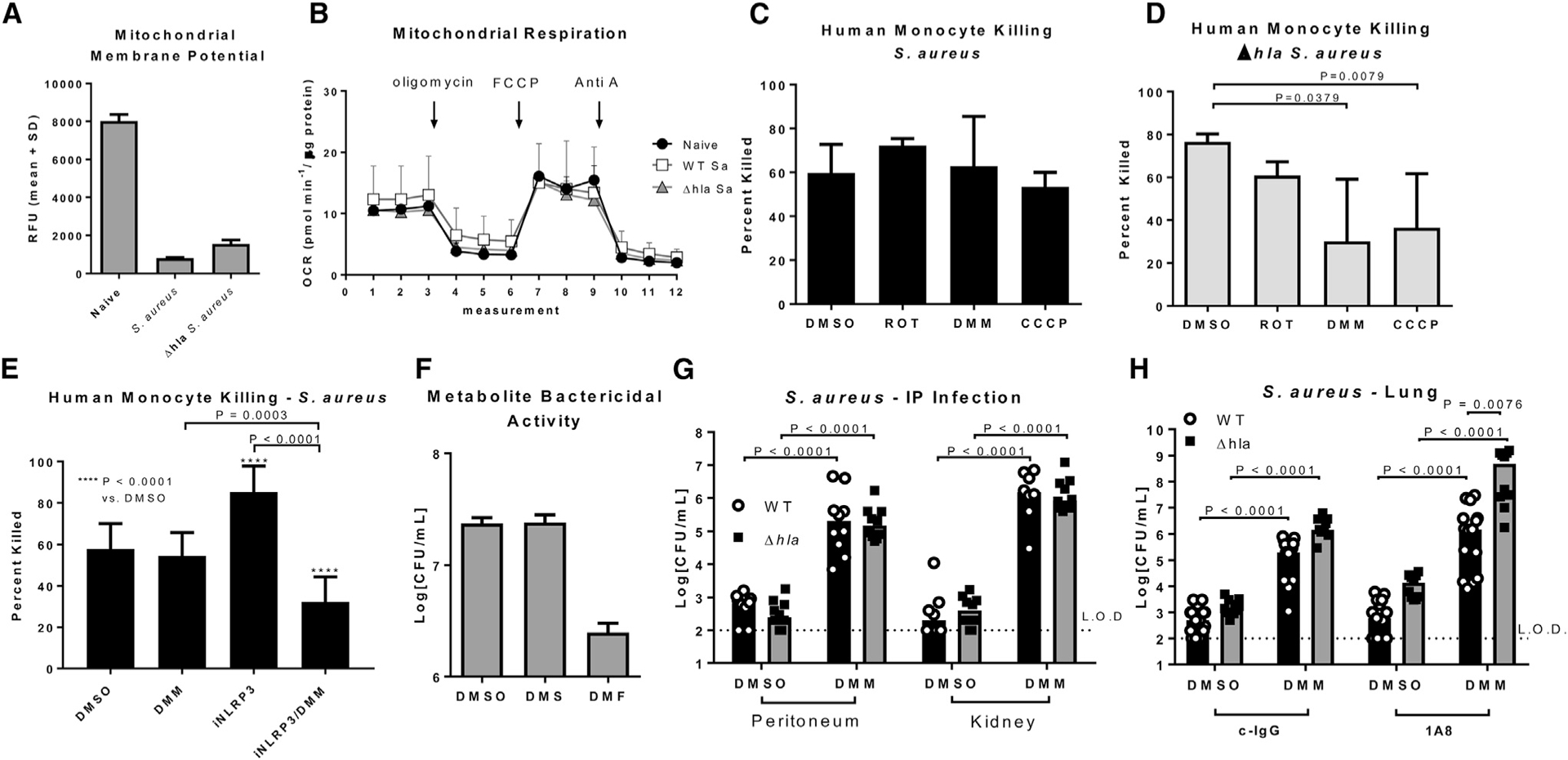
ETC Complex II Activity Contributes to the Killing of Δhla *S. aureus* (A) TMRM analysis of the mitochondrial membrane potential in naive primary human monocytes, or monocytes incubated (MOI 1) with WT or Δ*hla S. aureus*. (B) Seahorse analysis of mitochondrial respiration in naive primary human monocytes, or monocytes incubated (MOI 1) with WT or Δ*hla S. aureus*. (C) Percentage of *S. aureus* killed following 1-hr incubation with primary human monocytes treated (2 hr) with rotenone (ROT), DMM, CCCP, or DMSO. (D) Percentage of Δ*hla S. aureus* killed following 1-hr incubation with primary human monocytes treated (2 hr) with rotenone (ROT), DMM, CCCP, or DMSO. (E) Percentage of *S. aureus* killed following 1-hr incubation with primary human monocytes treated (2 hr) with DMM, iNLRP3, or both DMM and iNLRP3. (F) Numbers of *S. aureus* CFUs recovered following 3-hr incubation in PBS containing DMSO, diethyl succinate, or dimethyl fumarate. (G) Numbers of *S. aureus* CFUs recovered from the peritoneum and kidneys of mice infected (i.p., 1e6 CFUs) with WT or Δ*hla S. aureus* along with 600 mg/kg DMM. (H) Numbers of *S. aureus* CFUs recovered from the lungs of mice treated with 600 mg/kg DMM or DMSO (i.p., time of infection) and infected (IN, 1e6 CFUs) with WT or Δ*hla S. aureus*. Statistical significance (C–E and H) was determined by ANOVA followed by Dunn’s test or (G) Mann-Whitney test. All data are representative of at least 2 independent experiments. (A–F) n ≥ 4 replicates per group. Data presented as mean ± SD.

In response to live *Escherichia coli*, human monocytes upregulate complex II in the electron transport chain (ETC), which is necessary for antimicrobial responses. Increased succinate dehydrogenase (SDH, ETC complex II) activity results in increased mitochondrial ROS, Hif-1 stabilization, and elevated pro-IL-1β levels (Mills et al., 2016), and it is also correlated with a drop in mitochondrial membrane potential as ETC complexes I, III, and IV are the primary drivers of membrane voltage potential. To determine if similar changes in the ETC occur in response to *S. aureus*, we measured the effect of WT and Δ*hla S. aureus* on mitochondrial membrane potential using tetramethylrhodamine, methyl ester (TMRM) fluorescence. Both WT and Δ*hla S. aureus* reduced TMRM fluorescence, indicating that mitochondrial membrane potential drops in response to *S. aureus*, and these alterations in membrane potential were AT independent (Figure 4A). Unlike *E. coli* infection, neither WT nor Δ*hla S. aureus* affected mitochondrial respiration (Figure 4B).

ETC alterations, in complex II specifically, are required for defense against *E. coli* as mentioned previously. To test if ETC complex II is involved in bactericidal activity against *S. aureus*, we treated monocytes with rotenone (complex I inhibitor), dimethyl malonate (DMM) (complex II inhibitor), or carbonylcyanide m-chlorophenylhydrazoe (CCCP) to uncouple the ETC, and we monitored mitochondrial ROS production, caspase-1 activation, and bacterial killing. CCCP and DMM, not rotenone, reduced mitochondrial ROS and caspase-1 activity in both WT and Δ*hla S. aureus*-infected cells (Figures S5A and S5B). Human monocyte killing of WT *S. aureus* was not affected by any treatment (Figure 4C); however, Δ*hla S. aureus* killing was significantly reduced upon DMM (p = 0.0379) and CCCP (p = 0.0079) treatment, but not by rotenone (Figure 4D), confirming that ETC complex II is involved in antimicrobial functions.

We hypothesized that ETC complex II inhibition did not influence killing of WT *S. aureus* due to NLRP3-dependent physical dissociation of mitochondria from phagocytosed bacteria. Therefore, we tested the ability of human monocytes to kill WT *S. aureus* in the presence of DMM, MCC950, or a combination of DMM and MCC950, as inhibiting NLRP3 should sensitize monocytes to the effect of DMM. As before, we observed no effect of DMM on the killing of WT *S. aureus*, and MCC950 significantly increased bacterial killing (p < 0.0001) (Figure 4E). Treating cells with the combination of MCC950 and DMM significantly reduced killing as compared with DMSO control (p < 0.0001), DMM (p = 0.0003), and MCC950 alone (p < 0.0001), confirming that NLRP3 inflammasome activation uncouples mitochondria from the killing of *S. aureus*. Additionally, the product of succinate oxidation by ETC complex II, fumarate, is bactericidal to *E. coli* and *Salmonella enterica*, and it could provide a second antimicrobial pathway involved in mitochondrial-dependent *S. aureus* killing. Incubation of *S. aureus* with dimethyl fumarate, but not PBS or diethyl succinate, reduced *S. aureus* CFUs, indicating it was bactericidal to this pathogen as well (Figure 4F). Together, these data support the conclusions that AT prevents mitochondrial engagement in the bacterial killing process but, in the absence of AT, ETC complex II activity enhances bacterial killing.

To demonstrate complex II-driven mitochondrial involvement in bacterial killing *in vivo*, we intraperitoneally (i.p.) infected mice with 1e6 CFUs *S. aureus* or Δ*hla S. aureus* while simultaneously delivering DMM (600 mg/kg i.p.). Significantly increased numbers of WT and Δ*hla S. aureus* (p < 0.0001) were recovered from the peritoneum and kidney in mice treated with DMM, suggesting that complex II is required for host defense *in vivo* (Figure 4G). Due to the effect of DMM on the clearance of WT *S. aureus*, which suggests it also acts on cells independently of AT, we tested its ability to reduce killing of *S. aureus* by neutrophils, an AT-insensitive cell population. *In vitro* DMM reduced *S. aureus* killing by human neutrophils, confirming a more broad effect *in vivo* (Figure S5C). To directly test DMM’s effect on alveolar macrophages, we depleted neutrophils by i.p. injection of anti-Ly6G antibody (500 mg/kg, i.p.), or control IgG, 24 hr prior to intranasal infection with 1e5 CFUs of WT or Δ*hla S. aureus*. This innocula was chosen such that depletion of neutrophils does not significantly influence bacterial clearance (p = 0.5764), suggesting that resident immune cells, primarily alveolar macrophages, are responsible for bacterial killing (Figure 4H). Treatment with DMM (i.p.) at the time of infection significantly impaired clearance from the lungs of both control (p < 0.0001) and neutrophil-depleted (p < 0.0001) mice, demonstrating that ETC complex II is necessary for host defense by resident immune cells in the lung. Notably, DMM had a significantly larger effect (p = 0.0076) clearance of Δ*hla S. aureus* as compared with WT *S. aureus* in mice lacking neutrophils, reflecting the AT-dependent dissociation of mitochondria from bacterial killing in the alveolar macrophage.

## DISCUSSION

The durability of *S. aureus* as a major threat to humans is in part due to growing resistance to common frontline antibiotics, but also it is due to the wide array of virulence factors the bacteria utilize for immune evasion, persistence, and to damage host tissues, which can also potentiate other bacterial infections (Cohen et al., 2016). In the lung, AT activity has been described as a significant correlate of clinical disease severity, with potent activity on resident macrophages and the epithelial lining of the lung (Inoshima et al., 2011; Kebaier et al., 2012; Stulik et al., 2014). Murine studies identified NLRP3 inflammasome activity as the primary driver of AT-induced tissue damage and mortality, however, the mechanisms through which NLRP3 inflammasome activity promotes infection are not fully defined (Kebaier et al., 2012; Parker and Prince, 2012). Herein we show that inflammasome-dependent cytokine production does not impair bacterial clearance, but rather we find that AT-driven inflammasome activation disrupts mitochondrial-dependent antimicrobial processes within macrophages.

Alveolar macrophages play a central role in host defense against inhaled bacterial pathogens. Acting as the first line of defense against respiratory pathogens, these cells directly kill bacteria as well as coordinate the activation of other innate immune cell populations. Depletion of alveolar macrophages has been shown to impair clearance of multiple species of bacteria, including *S. aureus* (Cohen et al., 2016; Kitur et al., 2015; Martin et al., 2011). AT preferentially targets alveolar macrophages, as compared to recruited neutrophils, due to increased expression of ADAM10 on the macrophage. AT pore formation limits endosomal acidification, a process required for the killing of *S. aureus*, as well as other co-infecting opportunistic pathogens (Bidani et al., 2000; Cohen et al., 2016; Dey and Bishayi, 2015). We now show that AT-mediated impairment of phagosomal acidification and bacterial killing correlates with a physical separation of mitochondria and phagocytosed *S. aureus*.

The contribution of the mitochondria to inflammasome signaling and antimicrobial responses has recently been described (Garaude et al., 2016; Groß et al., 2016; Mills et al., 2016; West et al., 2011; Zhou et al., 2011). Mitochondria act as a scaffold on which NLRP3, caspase-1, and ASC are formed into the inflammasome complex in a MAVS-dependent manner (Bryan et al., 2009; Iyer et al., 2013; Subramanian et al., 2013). Mitochondrial ROS facilitates NEK7 binding to NLRP3, which is required for inflammasome activation (He et al., 2016; Shi et al., 2016). Additionally, mitochondria are recruited to bacteria-containing endosomes, in part by kinases Mst1 and Mst2, which facilitate mitochondrial movement through the GTPase Rac, and assembly of a protein complex consisting of Traf6 and mitochondrial complex I assembly factor ECIST (Geng et al., 2015). Clinically, a premature termination mutation in Mst1 is correlated with increased rates of infection, including some caused by *S. aureus* (Abdollahpour et al., 2012; Nehme et al., 2012). Our data confirm that Mst1 and Mst2 are required for maximal killing of *S. aureus*, although this occurs in both an AT-independent and -dependent fashion. These pathways are not unique to macrophages, as DMM also inhibited killing of *S. aureus* by neutrophils. As neutrophils lack the receptor for AT, ADAM10, *S. aureus* does not protect itself from neutrophil-mediated killing through the mechanisms described in this communication. How ETC complex II contributes to neutrophil-mediated killing remains to be fully elucidated. As DMM is not toxic and can actually protect in certain disease models, our data suggest a more global role for mitochondria in the innate immune response than just macrophage phagocytic killing (Chouchani et al., 2014).

In response to bacterial pathogen-associated molecular patterns (PAMPS), the ETC is altered resulting in increased mitochondrial ROS, caspase-1 activation, and production of the anti-microbial metabolite fumarate (Garaude et al., 2016). We find that *S. aureus* takes advantage of AT-mediated inflammasome activation to disengage mitochondria from these antimicrobial functions. When infected with Δ*hla S. aureus*, mitochondria co-localize with internalized bacteria, and mitochondrial ROS/caspase-1 and fumarate production result in eradication of the pathogen. In the presence of AT and activation of the NLRP3 inflammasome, mitochondria fail to associate with bacteria. Under these conditions, cells are unable to fully eliminate internalized bacteria, while inflammasome-dependent cytokines contribute to tissue inflammation. The importance of mitochondrial localization is reflected by the greater effect of caspase-1 neutralization on the killing of Δ*hla S. aureus* as opposed to WT *S. aureus*. This difference also explains the large caspase-1 effect reported by Sokolovska et al. (2013), in which the low AT-producing Reynolds strain was used (Figure S5A). Of note, killing of the Reynolds strain was also DMM sensitive (Figure S5B). We conclude that AT binding to the surface of ADAM10-expressing cells distracts the phagocyte from its primary role of bacterial eradication, providing a niche in which the pathogen can survive.

Inflammasome-knockout mice have long been appreciated to exhibit an improved capability for *S. aureus* clearance from the lung (Kebaier et al., 2012). Despite convincing evidence pointing to a detrimental role of NLRP3 in respiratory host defense against *S. aureus*, efforts to describe the contribution of inflammasome cytokines IL-1β and IL-18 have not resulted in a clear phenotype. Furthermore, neutralization of these cytokines has yet to result in benefits comparable to deletion of the inflammasome itself (Labrousse et al., 2014; Parker and Prince, 2012; Perret et al., 2012; Robinson et al., 2013). Our data support these findings, and while neutralization of either cytokine results in a small increase in survival, protection afforded by AT neutralization or NLPR3 inhibition is considerably higher. Furthermore, cytokine neutralization does not improve bacterial clearance when mice are infected with sublethal numbers of bacteria, demonstrating that protection afforded by NLRP3 inhibition is due to improved bacterial killing by macrophages rather than decreased levels of pro-inflammatory cytokines.

AT is a significant virulence factor used by *S. aureus*, however, *S. aureus* also expresses additional toxins, including PVL and leucocidin A/B, that can kill human cells at sufficient concentrations (Melehani et al., 2015). Human cells are more sensitive than murine cells to the activity of these additional toxins; however, herein we show that killing of *S. aureus* by primary human monocytes is significantly influenced by the deletion of AT, despite expression of PVL and leucocidin A/B (LukA/B). While monocytes are not a perfect match for fully differentiated alveolar macrophages, our data are consistent with data from studies utilizing a humanized mouse model of pneumonia, whose lung is patrolled by human alveolar macrophages, in which neutralization or deletion of PVL did not affect bacterial clearance to the same degree as AT neutralization (Cohen et al., 2016; Prince et al., 2017). While we cannot eliminate a role for these toxins in sublethal infection, these data suggest that AT plays a greater role in this model.

Mitochondria are increasingly appreciated as a crucial component of innate immune responses to bacterial pathogens. We now report that *S. aureus* evolved a mechanism to dissociate mitochondria from the antimicrobial response of the macrophage. Depletion of AT re-engages mitochondria in the process of bacterial killing. Clinically, neutralization of the AT, or inhibition of the inflammasome, could provide benefit against *S. aureus* infection by re-engaging macrophages in the defense against this significant human pathogen.

## EXPERIMENTAL PROCEDURES

### Institutional Approvals and Oversight

All animal studies were approved by the MedImmune Institutional Animal Care and Use Committee, and they were conducted in an Association for Accreditation and Assessment Laboratory Animal Care (AAALAC)-accredited facility in compliance with U.S. regulations governing the housing and use of animals. Use of human samples was approved by the MedImmune Institutional Review Board.

### *In Vivo* Models

Frozen stock cultures of *S. aureus* USA300 strain SF8300 were thawed and diluted to the appropriate inoculum in sterile PBS (pH 7.2) (Invitrogen) (Hua et al., 2014). Specific pathogen-free 7- to 8-week-old female C57BL/6J mice (The Jackson Laboratory) were briefly anesthetized and maintained in 3% iso-flurane (Butler Schein Animal Health) with oxygen at 3 L/min and infected intra-nasally. All bacterial suspensions were administered in 50 μL PBS. In select experiments, MEDI4893* c-IgG was administered in 0.5 mL i.p. 24 hr prior to infection. Anakinra (Kinaret, SOBI) was administered (10 mg/kg) i.p. for 4 days and the animals were infected on day 4, as previously described (Cohen and Prince, 2013). Neutralizing antibody against IL-18 (clone YIGIF74–1G7, Bio X Cell) or control Rat IgG2a was administered (15 mg/kg) i.p. 24 hr prior to infection. NLRP3 inhibitor MCC950 was delivered i.p. (50 mg/kg) in 0.5 mL PBS 1 hr prior to infection. Neutrophils were depleted by i.p. injection of anti-Ly6G antibody (500 mg/kg, Bio X Cell) as described previously (Cohen et al., 2016, 2017). For i.p. infections, *S. aureus* was prepared in sterile PBS and injected along with DMSO or DMM (600 mg/kg) in a 0.5 mL bolus (1e6 CFUs per animal). Animals were euthanized with CO_2_ at the indicated time points, and lung tissue, i.p. wash, or kidneys were collected for analysis. The bacterial load in lung, i.p. wash, and kidneys was determined by plating serial dilutions on tryptic soy agar (TSA).

### Cytokine Expression and BAL Protein

Mice were infected and dosed as described above. At 4 or 24 hr post-infection, groups of 5 animals were euthanized, and the BAL fluid was collected. BAL fluid (BALF) was spun in a micro-centrifuge at 500 × *g* for 5 min to remove cellular debris. The supernatants were aliquoted and stored at −80°C until as-sayed. IL-1β levels were quantitated using the Mouse IL-1β Ready-Set-Go ELISA kit (eBioscience). Protein concentrations were quantified using the Pierce 660nm Protein Assay (Thermo Fisher Scientific).

### Alveolar Macrophage Gene Expression

Alveolar macrophages were purified from the BAL of naive and infected mice by CD11c bead selection (STEMCELL Technologies). Cells purified from 10 mice were pooled and treated as a single sample for analysis. Total RNA was extracted using RNeasy Mini Kit (QIAGEN), following the manufacturer’s protocol, and it was treated with RNase-free DNase I to remove genomic DNA contamination. RNA was quantified using a NanoDrop 2000 spectrophotometer (Thermo Fisher Scientific), and the quality of RNA was assessed using the Agilent RNA ScreenTape assay in conjunction with a 4200 TapeStation system (Agilent Technologies). Only high-quality RNA samples with an RNA Integrity Number (RIN) greater than 9 were used for microarray hybridization.

RNA samples were amplified and labeled using MessageAmp Premier RNA Amplification Kit (Thermo Fisher Scientific), according to the manufacturer’s recommendations. Briefly, 75 ng total RNA was reverse-transcribed to first-strand cDNA with T7-oligo(dT) primer using ArrayScript reverse transcriptase, followed by second-strand cDNA synthesis to generate double-stranded cDNA (ds-cDNA). Subsequently, the ds-DNA was used as a template for *in vitro* transcription to synthesize biotin-labeled antisense RNA (aRNA) molecules. The biotin-labeled aRNA was purified with RNA-binding beads and then fragmented at 94°C for 35 min in fragmentation buffer (40 mM Tris-acetate [pH 8.2], 100 mM Potassium Acetate, and 30 mM Magnesium Acetate). Fragmented aRNA (10 μg) was hybridized to Affymetrix GeneChip Mouse Genome 430 2.0 Array (Thermo Fisher Scientific) at 45°C for 18 hr. Affymetrix GeneChip Fluidics Station 450 was used for washing and staining of the arrays, and hybridized arrays were scanned using a GeneChip Scanner 300 7G (Thermo Fisher Scientific), according to the manufacturer’s user guide. Array files were analyzed using packages from R Bioconductor: normalization was performed using the robust multi-array average (RMA) method implemented in affy package; probe IDs were converted into Entrez gene IDs and filtered to interquartile range (IQR) = 0.33 using method nsFilter in genefilter package (Gentleman et al., 2019); analysis for differential gene expression was performed with limma package; ranking of genes from limma analysis was used as input to gene set enrichment analysis method gsePathway implemented in ReactomePA package (Yu and He, 2016); per-sample pathway activities for the heatmap visualization were estimated with gene set variation analysis (GSVA) package (Hänzelmann et al., 2013); heatmaps were shown with ComplexHeatmap package (Gu et al., 2016); and MGSAT R software was used to implement the entire analysis pipeline (Gautier et al., 2004; Luo et al., 2009; Ritchie et al., 2015; Tovchigrechko, 2017).

### Bacteria-Killing Assay

BMDMs were generated from bone marrow isolated from the femurs and tibias of naive mice. BMDMs were cultured for 7 days in RPMI-1640 supplemented with 10% fetal bovine serum (FBS) and 20 ng/mL macrophage colony stimulating factor (m-CSF). Human peripheral blood monocytes (CD14^+^) were isolated from blood donated by healthy, anonymous volunteers (MedImmune employees, male and female) using CD14 microbeads (Miltenyi Biotec). BMDMs or human peripheral blood monocytes were plated (200,000 cells/96 wells). Cells were washed with antibiotic-free media and incubated with *S. aureus* (MOI 0.1, killing assays; MOI 10, phagocytosis assays). In select experiments, cells were pretreated (2 hr) with caspase I inhibitor (ac-YVAD-cmk, 10 μM, Calbiochem), NLRP3 inhibitor (MCC950, 15 nM, Cayman Chemical), NOX1/4 inhibitor (GKT137831, 150 nM, Cayman Chemical), or mitoTEMPO (100 μM, Sigma-Aldrich) or equivalent volume DMSO control. Bacteria were prepared in the same manner as those for *in vivo* studies. To measure phagocytosis, gentamycin (300 μg/mL) was added to the media 1 hr following bacterial inoculation, incubated 30 min, the cells were washed with clean PBS, the cells were lysed with Triton X-100 (0.1% in PBS), and the bacterial CFUs were enumerated by serial dilution. To measure killing, the entire contents of the well (cells and media) were removed at time 0 and 1 hr, and bacterial CFUs were enumerated by serial dilution.

### siRNA Transfection

BMDMs were transfected with ON-TARGETplus SMARTpool containing 4 siRNA targeting *Nlrp3*, *Pycard*, or *Mst1/2* (Dharmacon) or control siRNA with Lipofectamine 2000 (Thermo Fisher Scientific). Briefly, 1e5 BMDMs were plated in each well of a 24-well plate in complete media 24 hr prior to infection. siRNA was diluted in Opti-MEM without serum (500 nM) and complexed with Lipofectamine according to the manufacturer’s instructions. siRNA complex (50 μL) was added to each well and incubated for 3 days, at which point the cells were used for either bacteria-killing assays or imaging studies.

### Bacterial pH Measurement

SF8300 *S. aureus* was labeled by incubation with fluorescein isothiocyanate (FITC) Isomer I and Alexa Fluor 647 NHS Ester (Succinimidyl Ester) (2.5 mg/mL, 1 hr, Life Technologies). Bacteria were washed three times in fresh PBS to remove unbound dye and frozen until use. BMDMs or human peripheral blood monocytes were prepared as described above and incubated with bacteria at MOI 1. Following 1-hr incubation, the cells were placed on ice, washed with cold PBS to remove free bacteria, and scraped from the well. Acidificatin of the bacterial microenvironment was determined by measuring FITC intensity (maximum fluorescence intensity [MFI]), which is reduced in acidic environments, in cells containing bacteria as described previously (Cohen et al., 2016).

### Confocal Imaging

*S. aureus* was labeled with Calcein Blue, AM (5 μM, Thermo Fisher Scientific) as previously described (Cohen et al., 2016). BMDMs or primary human monocytes were treated as described above. In select experiments, mitochondria were labeled with Mitotracker CMXRos (Molecular Probes) prior to incubation with bacteria. The cells were incubated with labeled *S. aureus* at MOI 1 in a 96-well plate. For caspase-1 imaging in cells, FAM-FLICA (Immunochemistry Technologies) was added to each well 1 hr post bacterial inoculation, and cells were stained in accordance with the manufacturer’s instructions. The cells were washed 3 times in fresh PBS prior to imaging, and 20 μL cell suspension was loaded onto a coverslip. The cells were maintained at 37°C and 5% CO_2_ throughout the imaging process. To image ASC, cells were allowed to adhere to coverslips overnight, prior to labeling mitochondria and infecting as described above. Following a 1-hr incubation with bacteria, cells were fixed with 10% formalin (10 min), permeabilized with 0.5% Triton X-100 in PBS (10 min), blocked with BlockAid (Thermo Fisher Scientific, 1 hr), and stained overnight with anti-ASC/TMS1 (Novus Biologicals). ASC/TSM1 was visualized with AF647 conjugated anti-rabbit IgG secondary (Molecular Probes). The slides were imaged using a Leica TCS SP5 X confocal microscope (Leica Microsystems).

### Measurement of Mitochondrial ROS, Mitochondrial Membrane Potential, and Caspase-1 Activation

Human peripheral blood monocytes were incubated with mitoSOX (1 hr prior to bacterial incubation) (Molecular Probes), tetramethylrhodamine, methyl ester (TMRM 20 nM, 1 hr post bacterial incubation) (Mills et al., 2016), or FAM-FLICA (1 hr post bacterial incubation). Fluorescence intensity was measured using the LSR II Flow Cytometer (BD Biosciences) and analyzed with FlowJo.

### AT Quantification

AT quantification was performed from isolate culture supernatant as previously described (Sharma-Kuinkel et al., 2015). Briefly, a single colony from a fresh TSA plate of each strain was inoculated in 3 mL tryptic soy broth (TSB; Becton Dickinson) and incubated overnight at 37°C under 220 rpm. The appropriate amount of overnight culture corresponding to optical density 0.1 at 600 nm (OD600) was inoculated into 10 mL TSB in a 150-mL flask and incubated at 37°C under 220 rpm. After 16 hr, the culture was spun down at 3,100 rpm at 4°C for 15 min to remove the pellets. The supernatant was then filter-sterilized and transferred to a sterile tube and stored at −80°C. 96 well high-bind plates (VWR International) were coated overnight at 4°C with anti-AT mAb MEDI4893 (0.1 μg/mL) in 0.2 M carbonate/bicarbonate buffer. Following washing with PBS/0.1% tween 20 (PBST), plates were blocked with PBST+ 5% BSA for 1 hr at room temperature (RT). Overnight *S. aureus* culture supernatants were serially diluted 2-fold starting at a 1:100 dilution, and a purified AT reference standard was added and incubated at RT for 1 hr. The plates were washed 4 times with PBST, incubated for 1 hr with an anti-AT rabbit IgG (1 mg/mL), and the rabbit IgG detected with an HRP-conjugated goat anti-rabbit IgG (1:10,000) (Jackson ImmunoResearch Laboratories). Following a 1-hr incubation at RT, the plates were washed 4 times with PBST, and antibody binding was detected with 100 μL SureBlue TMB substrate (KPL), followed by neutralization with 100 μL TMB Stop Solution (KPL). OD_450_ was measured on a Sunrise plate reader (Tecan).

### Western Blot

Cells were lysed with radioimmunoprecipitation assay (RIPA) buffer (Thermo Fisher Scientific) containing cOmplete protease inhibitor (Sigma) and frozen. Equal amounts of protein were separated on 4%–12% bis-Tris NuPage gels and transferred to polyvinylidene fluoride (PVDF) membranes (Thermo Fisher Scientific). Immunodetection was performed using anti-Mst1 (Cell Signaling Technology cat# 3682S), anti-Mst2 (Cell Signaling Technology cat# 3952S), and anti-actin (Sigma cat# A3854). Proteins were visualized with the Odyssey imaging system (LI-COR Biosciences).

### Mitochondrial Respiration

Mitochondrial oxygen consumption was measured using the Seahorse Xfe96 analyzer (Agilent Technologies). Primary human monocytes (1e6 cells per well) were incubated with *S. aureus* for 1 hr, after which the medium (DMEM containing 5 mM glucose, 4 mM L-glutamine, and 2 mM sodium pyruvate [pH 7.4]) was refreshed to remove any extracellular bacteria. The plate was placed in a CO_2_-free incubator for 1 hr prior to being placed in the analyzer. The following compounds were used in the mitochondrial stress test: 1 μM oligomycin (Sigma), 1 μM FCCP (Sigma), and 5 mM antimycin A (Sigma). The data represent the average of two independent experiments each with a minimum of 5 replicates.

### Electron Microscopy

Primary isolated islets were chemically fixed in 2% glutaraldehyde/4% para-formaldehyde in 0.1 M sodium cacodylate buffer at 4°C for 24 hr. After fixation, islets were stained with 1% osmium tetroxide in 0.1 M cacodylate buffer for 1 hr, rinsed, and then stained with 1% uranyl acetate for 1 hr in maleate buffer (pH 5.2). Islets were then rinsed and dehydrated via ethanol series, followed by embedding. The embedding resin consisted of 48% Embed 812, 31% dodecenyl succinic anhydride (DDSA), 21% methyl-5-norbornene-2,3-dicarboxylic anhydride (NMA), and 1.4% 2,4,6-tri[dimethyl-aminomethyl]phenol (DMP-30; Electron Microscopy Sciences, Hatfield, PA). Islets were incubated first with a 2:1 ratio of propylene oxide to embedding resin for 1 hr, then a 1:1 ratio for an additional hour, followed by 100% embedding resin overnight. The embedding resin containing islets was then polymerized overnight at 65°C in Electron Microscopy Sciences (EMS) silicone-rubber embedding molds. Once hard-ened, 80-nm sections were cut using a Leica UC6 ultramicrotome (Wetzlar, Germany), placed on EMS 200-mesh London Finder Formvar/carbon-coated copper grids stained with 2% uranyl acetate in 70% methanol and 0.5% lead citrate in distilled water, and then imaged at 2,500× using the FEI Tecnai G2 SPIRIT electron microscope equipped with a charge coupled device (CCD) camera (Pleasanton, CA) at 120,000 V. Images were acquired using the GATAN digital micrograph software.

### Statistical Analyses

Data were analyzed using t tests, Mann-Whitney tests, ANOVA followed by Dunnett’s test, Kruskal-Wallis followed by Dunn’s test, or log-rank test where appropriate. A Fisher’s exact test was used to compare rates of mitochondrial and bacterial association. All statistical analyses were performed using Graph-Pad Prism version 6.0. Sample sizes for all animal studies were estimated using log-rank test with 5% type I error rate and 80% power. The hypothesized effect size for each comparison was derived from historical data or pilot study data. Sample sizes were calculated using nQuery Advisor 7.0 software. All animals were randomly assigned to treatment groups using an online randomization tool implemented in Microsoft Excel. A p value of ≤ 0.05 was considered statistically significant.

## Supplementary Material

Supplemental fig 1-6

## Highlights

- Alpha toxin activates the inflammasome, preventing bacterial clearance
- NLRP3 activation, not downstream cytokine production, leads to impaired defense
- Macrophage NLRP3 activation sequesters mitochondria away from internalized *S. aureus*
- Mitochondrial ROS is required for caspase-1 activation and bacterial killing in macrophages
