# Supplementary material for: S. aureus Evades Macrophage Killing through NLRP3-Dependent Effects on Mitochondrial Trafficking
Source: Cell Rep. Author manuscript; Available in PMC 2020 Apr 16. (PMC7160668; doi:10.1016/j.celrep.2018.02.027)
Supplement: Supplemental fig 1-6 [file NIHMS1572922-supplement-Supplemental_fig_1-6.pdf]

Cell Reports, Volume *22*

Supplemental Information

S. aureus Evades Macrophage Killing through NLRP3-Dependent Effects on Mitochondrial Trafficking

Taylor S. Cohen, Michelle L. Boland, Brandon B. Boland, Virginia Takahashi, Andrey Tovchigrechko, Young Lee, Aimee D. Wilde, Mark J. Mazaitis, Omari Jones-Nelson, Christine Tkaczyk, Rajiv Raja, C. Kendall Stover, and Bret R. Sellman

S. aureus evades macrophage killing through NLRP3 dependent effects on mitochondrial trafficking

Taylor S. Cohen[1*], Michelle L. Boland[2], Brandon B. Boland[2], Virginia Takahashi[1], Andrey Tovchigrechko[1], Young Lee[3], Aimee D. Wilde[4], Mark J. Mazaitis[5], Omari Jones-Nelson[1], Christine Tkaczyk[1], Rajiv Raja[3], C. Kendall Stover[1], Bret R. Sellman[1]

[1] Department of Infectious Disease, [2] Department of Cardiovascular and Metabolic Disease, [3] Department of Translational Medicine and Pharmecogenomics, Medimmune LLC, Gaithersburg, MD

[4] Department of Pathology, Microbiology, and Immunology, Vanderbilt University Medical Center, Nashville, Tennessee

[5] Nascent Studio, Ann Harbor, MI

Conflict of Interest Statement:

All authors are employees of, or have received funding from, MedImmune, a member of the AstraZeneca group.

*Corresponding/Lead Author

One Medimmune Way
Gaithersburg MD 20878
cohent@medimmune.com

Supplemental Figures:

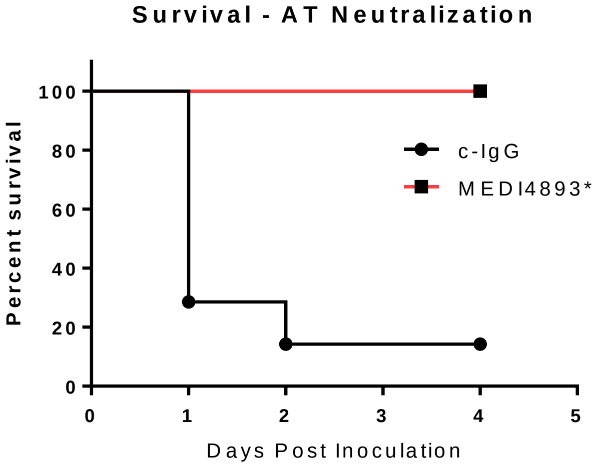

Supplemental Figure 1: AT neutralization improves survival. Related to Figure 1.

Survival of mice treated (24h prior to infection) with MEDI4893* or c-IgG. Representative of greater than 2 independent experiments, N ≥ 10 mice per group per experiment.

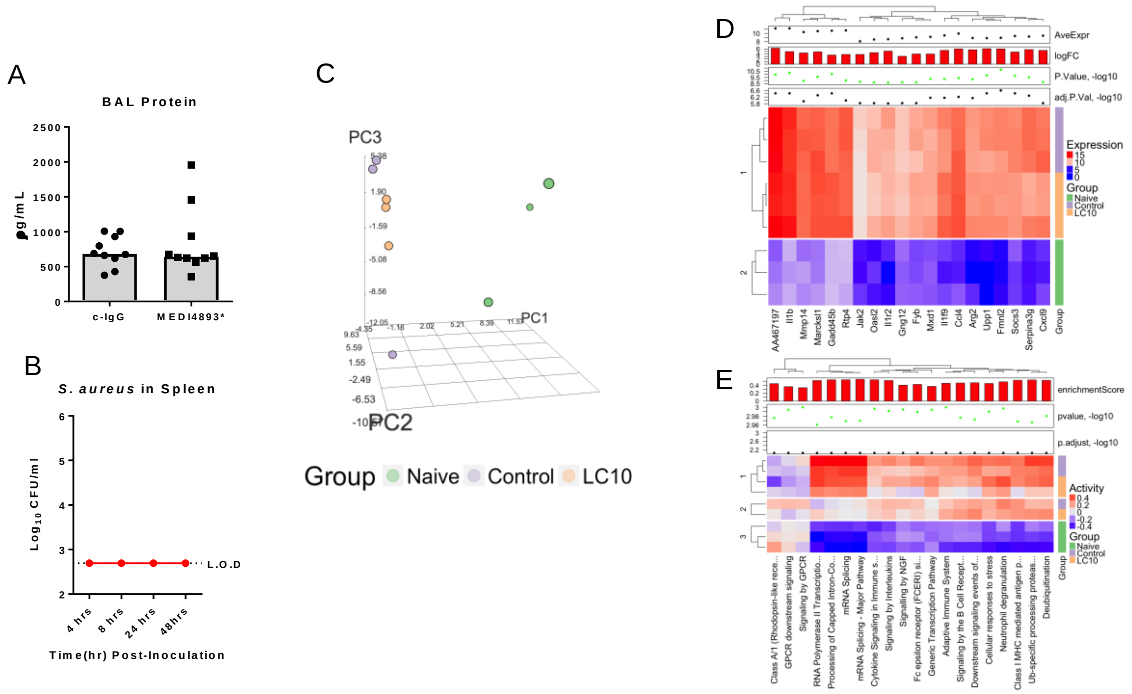

Supplemental Figure 2: Alpha toxin does not enable escape from the lung or alter AM gene expression. Related to Figure 2.

A, Protein levels in BALF of mice prophylactically treated with c-IgG or MEDI4893* and infected with *S. aureus* (5e7 CFU, 24h). **B**, *S. aureus* CFU recovered at the indicated time points from the spleens of mice infected with (5e7 CFU) *S. aureus*. **C**, Principle component analysis of gene expression in purified alveolar macrophages isolated from naïve (green) or infected mice prophylactically treated with c-IgG (orange) or MEDI4893* (LC10, purple). **D**, Heat map of top 20 genes differentially expressed between c-IgG and MEDI4893* (LC10) treated cells considered as a single group, and naïve cells. **E**, Heat map of top 20 differential pathways derived from the gene-level analysis shown in D. All data representative of 3 independent experiments. (**B-E**) N = 10 mice per time group.

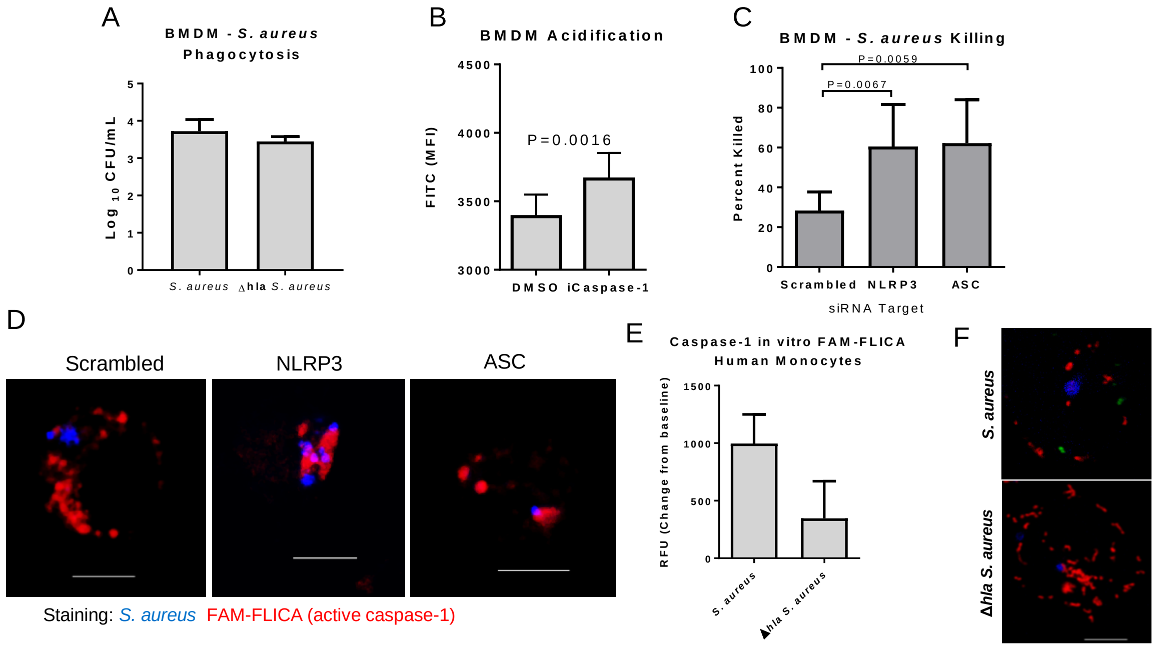

Supplemental Figure 3: AT influences bacterial killing via NLPR3 inflammasome activation. Related to Figure 2.

A, Numbers of *S. aureus* phagocytosed by BMDMs over a 1h incubation period. **B**, FACS analysis of acidification of the bacterial microenvironment in BMDMs infected with Δhla *S. aureus* in the presence of caspase-1 inhibitor or DMSO. **C**, Percent of *S. aureus* killed following 1h incubation with siRNA treated BMDMs (MOI 1). **D**, Confocal images of active caspase-1 (FAM-FLICA, red) and *S. aureus* (blue) within live siRNA treated BMDMs following 1h incubation with the bacteria. Scale bar 5 μm. **E**, Activation of caspase-1 in human monocytes 1h following infection with WT or Δhla *S. aureus*. **F**, Confocal images of ASC speck formation (ASC – green, *S. aureus* – blue, mitochondria – red). All data representative of 3 independent experiments. N ≥ 4 replicates per group.

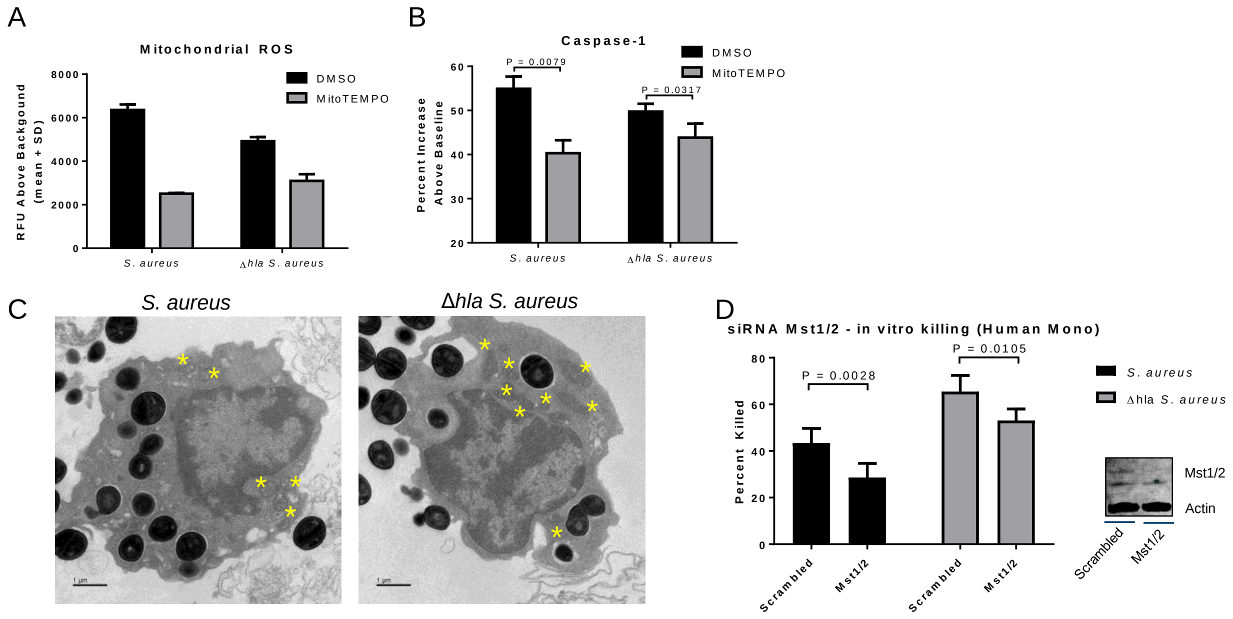

Supplemental Figure 4: MitoROS inhibition contributes to caspase-1 and bacterial killing. Related to Figure 3.

A, FACS analysis of mitochondrial ROS production in primary human monocytes treated with mitoTEMPO or DMSO and incubated with WT or Δhla *S. aureus* for 1h. **B**, FACS analysis of caspase-1 activation in primary human monocytes treated with mitoTEMPO or DMSO and incubated with WT or Δhla *S. aureus* for 1h. **C**, Electron micrographs of primary human monocytes incubated with WT or Δhla *S. aureus* for 1h. **D**, Percent of WT or Δhla *S. aureus* killed following 1h incubation with scrambled or *Mst1/2* siRNA treated primary human monocytes (MOI 1). Western blot confirming knockdown of Mst1 and Mst2 is shown in the inset. All data representative of 3 independent experiments. (**A**, **B**, **D**) $N \geq 4$ replicates per group. (**C**) representative image at least 5 images per experiment.

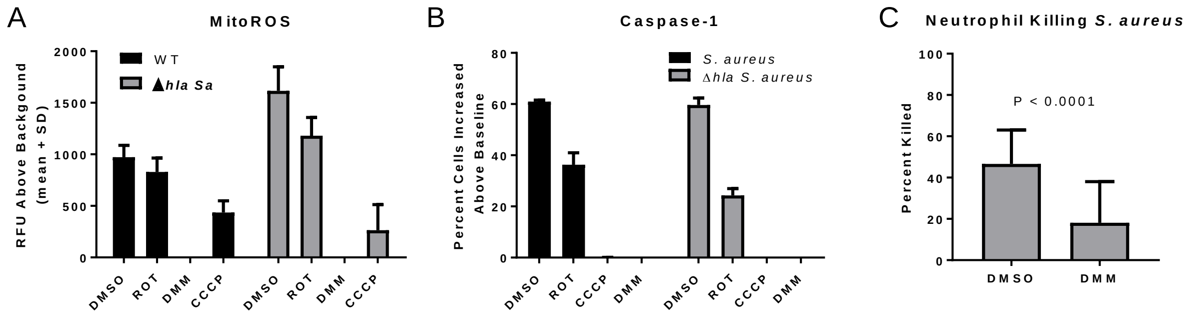

Supplemental Figure 5: ETC complex II inhibition prevents mitochondrial ROS and caspase-1 induction. Related to Figure 4.

A, FACS analysis of mitochondrial ROS production in primary human monocytes treated (2h) with rotenone (ROT), DMM, CCCP, or DMSO and incubated with WT or Δhla *S. aureus* for 1h. **B**, FACS analysis of caspase-1 activation in primary human monocytes treated (2h) with rotenone (ROT), DMM, CCCP, or DMSO and incubated with WT or Δhla *S. aureus* for 1h. **C**, Percent of *S. aureus* killed following 1h incubation with primary human neutrophils treated (2h) with DMM or DMSO. All data representative of at least 3 independent experiments. N ≥ 4 replicates per group.

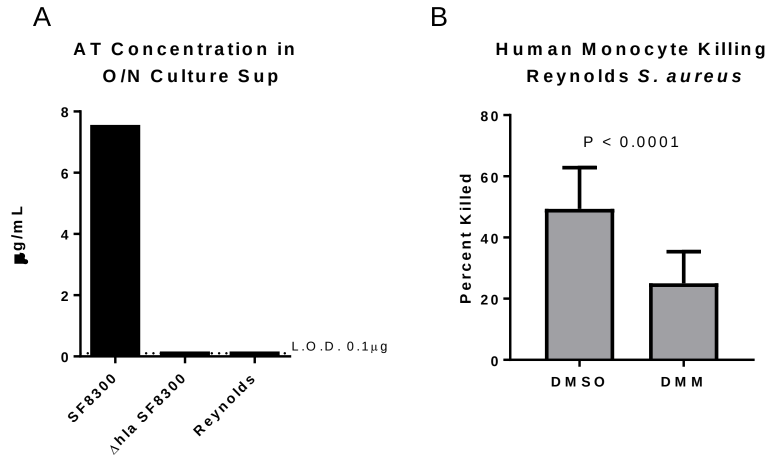

Supplemental Figure 6: AT production by SF8300 and Reynolds. Related to Figure 4.

A, Levels of AT produced by WT SF8300, Δhla SF8300 or Reynolds strains of *S. aureus*. **B**, Percent of *S. aureus* (Reynolds) killed following 1h incubation with primary human monocytes treated (2h) with DMM or DMSO. All data representative of at least 3 independent experiments. (**B**) N ≥ 4 replicates per group.
